# Pulsed-Field Ablation With Transcatheter Left Atrial Appendage Occlusion for Left Atrial Appendage–Origin Atrial Tachycardia

**DOI:** 10.1016/j.jaccas.2026.106879

**Published:** 2026-02-06

**Authors:** Junya Komatsu, Hiroki Sugane, Yuki Nishimura, Nao Okamoto, Hayato Hosoda, Yoko Nakaoka, Shinji Mito, Shuichi Seki, Kazuya Kawai

**Affiliations:** Department of Cardiology, Chikamori Hospital, Kochi, Japan

**Keywords:** left atrial appendage closure and catheter ablation, left atrial appendage isolation, pulse field ablation

## Abstract

**Background:**

Pulsed-field ablation (PFA) may be an emerging treatment for left atrial appendage (LAA)–origin atrial tachycardia (AT), but it may cause thrombus in the LAA. Concomitant transcatheter LAA occlusion (LAAO) may address this risk.

**Case Summary:**

A 54-year-old man with symptomatic LAA-origin AT underwent PFA. Post-PFA transesophageal echocardiography showed significant pectinate muscle edema with narrowing of the LAA orifice. To avoid late peridevice leak, a 40-mm Watchman FLX Pro (Boston Scientific) was implanted with intentional overcompression. At the 1-month follow-up, cardiac computed tomography demonstrated a complete LAA seal with adequate device compression.

**Discussion:**

Concomitant PFA and LAAO is feasible for LAA-origin AT. Because PFA can induce significant edema within the LAA, device selection based on pre-PFA imaging is critical to ensure durable sealing.

**Take-Home Messages:**

Careful pre-PFA imaging-based device sizing is critical when combining PFA with LAAO in patients with LAA-origin AT.

## Visual Summary

**Figure:**
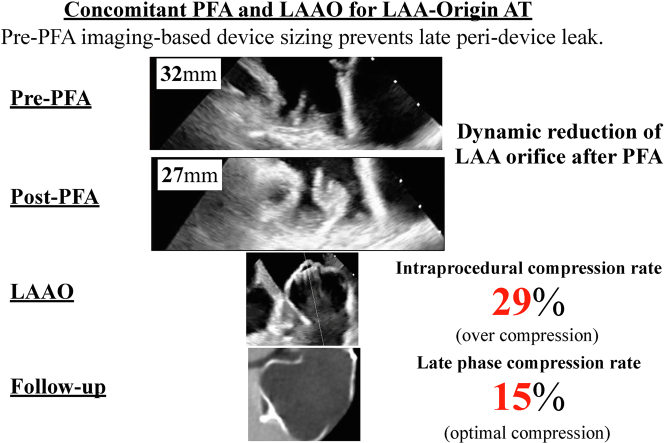

**Visual Summary:**
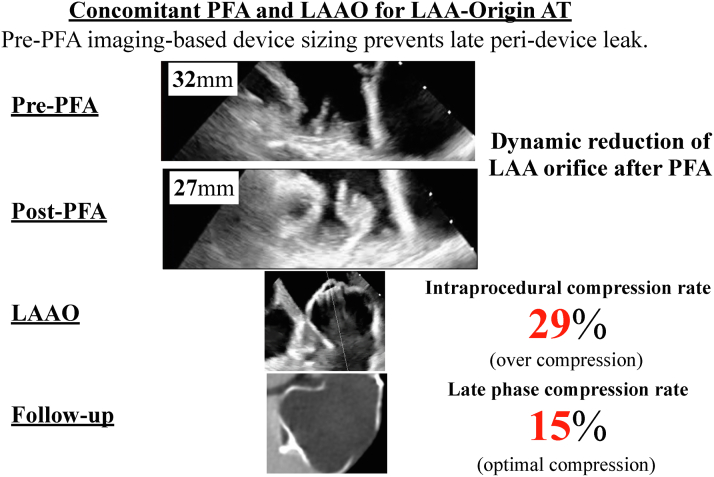
Concomitant PFA and Transcatheter LAAO for LAA-Origin AT As PFA can induce acute postablation LAA edema, device sizing based on preablation imaging is critical. AT = atrial tachycardia; LAA = left atrial appendage; LAAO = left atrial appendage occlusion; PFA = pulsed-field ablation.

## History of Presentation

A 54-year-old man presented to the outpatient office of our hospital with recurrent palpitations. On examination, his blood pressure was 120/80 mm Hg, his heart rate was 80 beats/min and regular, and there were no signs of heart failure. The arrhythmia terminated spontaneously, but he sought repeat catheter ablation to control symptoms.

## Past Medical History

Six months earlier, the patient had undergone standard radiofrequency catheter ablation (RFCA) for paroxysmal atrial fibrillation and the left atrial appendage (LAA)–origin atrial tachycardia (AT) arising from the distal part of the LAA.

## Investigations

**Figure 1:**
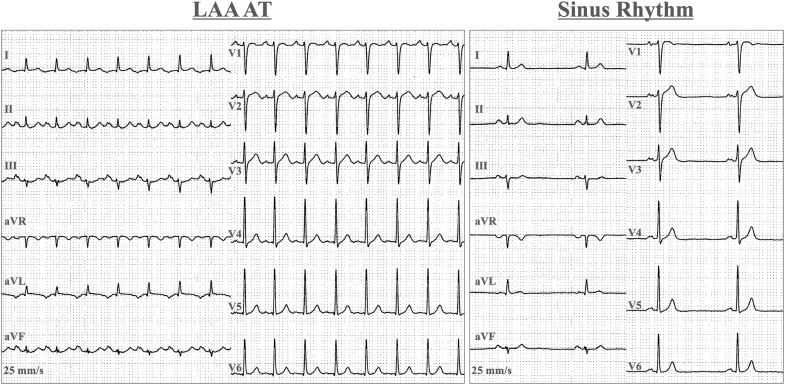
Index 12-Lead Electrocardiograms Electrocardiograms documenting LAA-origin AT and sinus rhythm for comparison. AT = atrial tachycardia; LAA = left atrial appendage.

**Figure 2:**
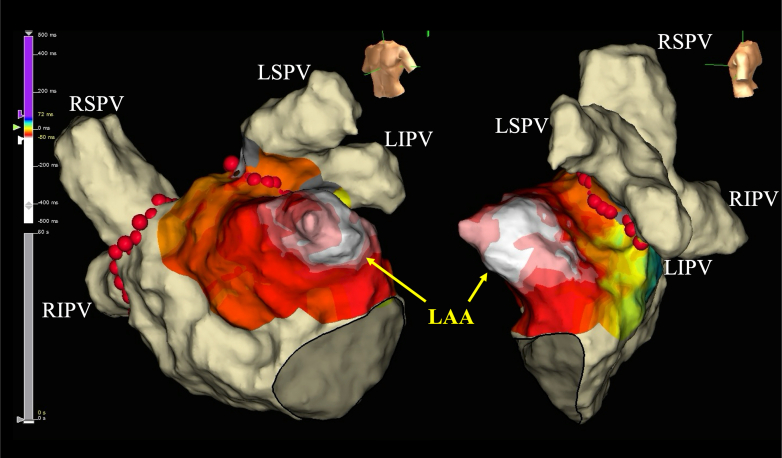
3-Dimensional Activation Mapping at the First Session Activation mapping identifying the earliest atrial activation within the LAA with centrifugal spread to the left atrium. LAA = left atrial appendage; LIPV = left inferior pulmonary vein; LSPV = left superior pulmonary vein; RIPV = right inferior pulmonary vein; RSPV = right superior pulmonary vein.

**Figure 3:**
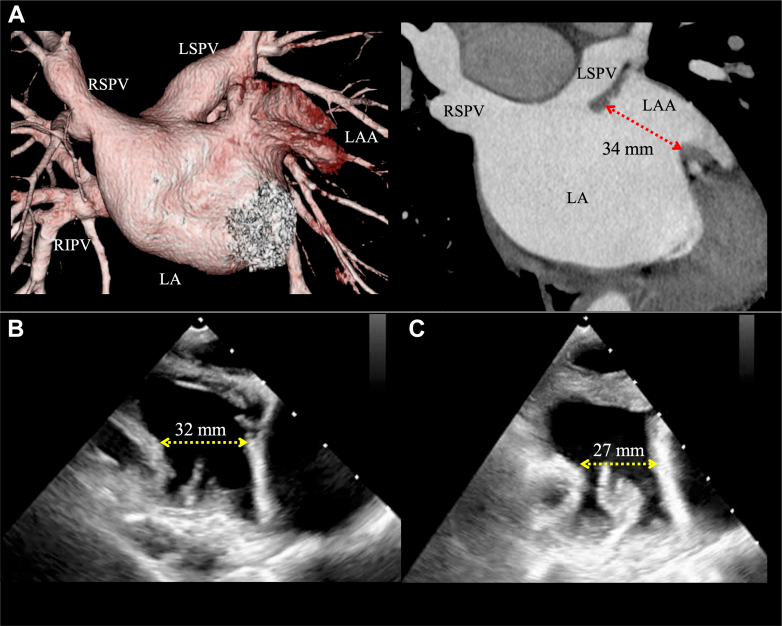
LAA Orifice Diameter Findings: Comparison Before and After PFA (A) Preprocedural cardiac CT demonstrating a cactus-shaped LAA with a maximum diameter of 34 mm. Intraprocedural TEE at a 135° angle view (B) before and (C) after PFA showed acute edema of the pectinate muscle with ostial narrowing and contour change caused by PFA, reducing the maximal diameter from 32 mm (B) to 27 mm (C). CT = computed tomography; PFA = pulsed-field ablation; TEE = transesophageal echocardiography; other abbreviations as in Figure 2.

A 12-lead electrocardiogram showed a positive P-wave deflection in lead V_1_, bifid (notched) P waves in leads II and V_1_, and an inverted P wave in lead I. These findings were suggestive of AT originating from either the left pulmonary vein or the LAA (Figure 1). Transthoracic echocardiography showed normal left ventricular systolic function without valvular or structural abnormalities. Laboratory tests, including thyroid function and cardiac biomarkers, were within normal limits. Ambulatory electrocardiogram monitoring documented frequent episodes of AT. At the initial ablation, invasive electrophysiology study with three-dimensional mapping at the first session showed complete electrical isolation of the pulmonary veins but incomplete suppression of the LAA-origin AT from the distal part of the LAA (Figure 2). Cardiac computed tomography (CT) demonstrated a cactus-shaped LAA with a maximum diameter of 34 mm without thrombus (Figure 3A).

## Differential Diagnosis

Alternative mechanisms of AT were considered, including focal AT from other atrial regions and atypical atrial flutter. However, the clinical history and the findings of a prior electrophysiology study with three-dimensional mapping strongly implicated recurrent LAA-origin AT.

## Management

Surgical LAA removal was discussed but was declined in favor of catheter-based treatment. Given the prior unsuccessful RFCA, concomitant pulsed-field ablation (PFA) and transcatheter LAA occlusion (LAAO) were planned. The procedure was performed under general anesthesia with transesophageal echocardiography (TEE) guidance. The pre-PFA TEE and the LAA angiography showed a large cactus-shaped LAA with a maximum orifice diameter of 32 mm and no thrombus (Videos 1 and 2). In accordance with this TEE imaging, a 40-mm Watchman FLX Pro (Boston Scientific) was considered appropriate for LAAO.

**Figure 4:**
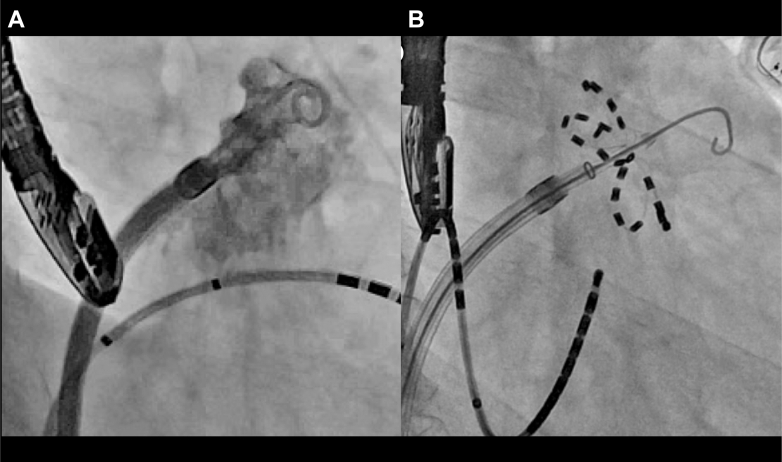
Findings on LAA Angiography (A) LAA angiography in the right anterior oblique 30° and caudal 30° views. (B) Fluoroscopic image during PFA at the same projection. LAA = left atrial appendage.

**Figure 5:**
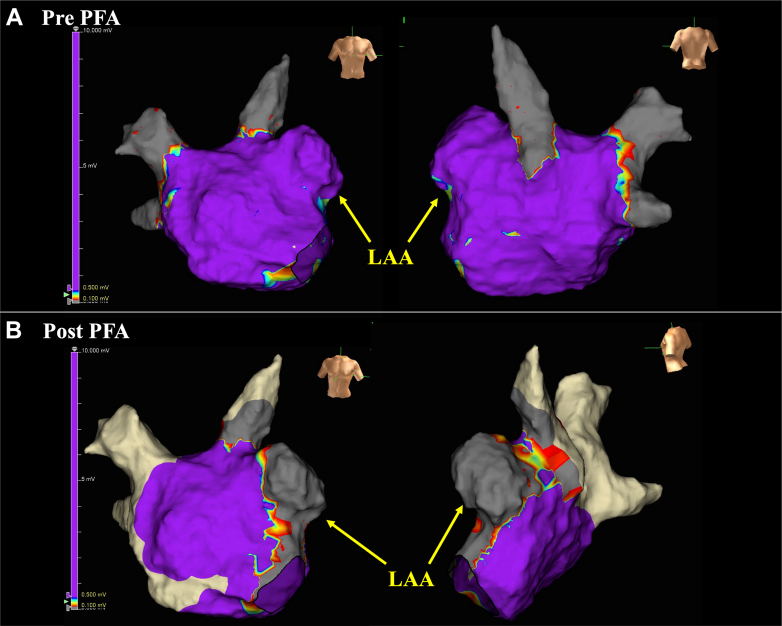
Left Atrial Voltage Mapping Before and After PFA (A) Pre-PFA left atrial voltage map showing preserved potentials across the LAA-ostial region. (B) Post-PFA voltage map demonstrating abolition of LAA potentials; entrance and exit block were confirmed by pacing maneuvers and lack of capture from within the LAA. LAA = left atrial appendage; PFA = pulsed-field ablation.

First, we tried to induce AT, but it was not induced before the procedure. Based on the clinical course, we believed the clinical AT was highly likely to originate from the LAA; we then achieved electrical LAA isolation using the Farapulse PFA system (Boston Scientific). After atrial transseptal puncture, a 12-F Farawave multielectrode pentaspline catheter (Boston Scientific) was advanced into the LAA. Multiple applications (2.5 seconds and 2 kV each) were delivered in the basket and flower configurations (Figure 4), resulting in successful electrical LAA isolation, as evidenced by local dissociation (Figure 5).

**Figure 6:**
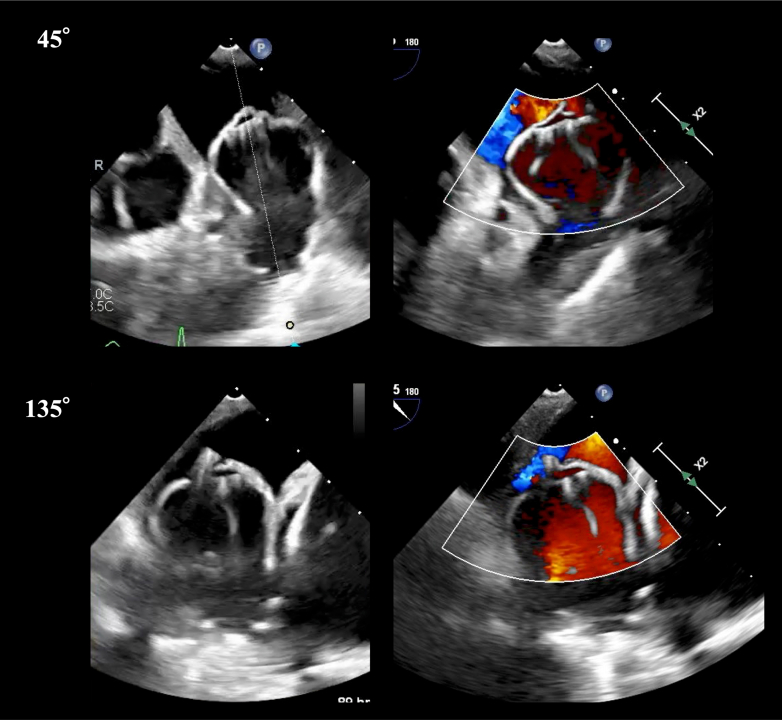
Intraprocedural TEE Findings During LAAO A 40-mm device was deployed according to the preablation plan with intentional overcompression (29%), yielding stable seating and no peridevice leak on color Doppler. LAAO = left atrial appendage occlusion; TEE = transesophageal echocardiography.

Subsequently, the Farapulse system was exchanged for a 15-F Watchman FXD Curve Access system (Boston Scientific) to perform LAAO. However, the post-PFA TEE revealed significant edema of the pectinate muscle, narrowing the LAA orifice from 32 to 27 mm (Figures 3B and 3C, Video 3). Downsizing of the occlusion device from 40 to 35 mm was considered, but this raised concern for late peridevice leak after the improvement of edema. Despite the swollen pectinate muscle occupying part of the LAA, deployment of a 40-mm device from the distal anterior lobe remained feasible (Figure 6). Therefore, a 40-mm Watchman FLX Pro was carefully deployed with intentional overcompression (compression rate: 29%), achieving stable positioning without complications (Videos 4 and 5).

## Outcome and Follow-Up

**Figure 7:**
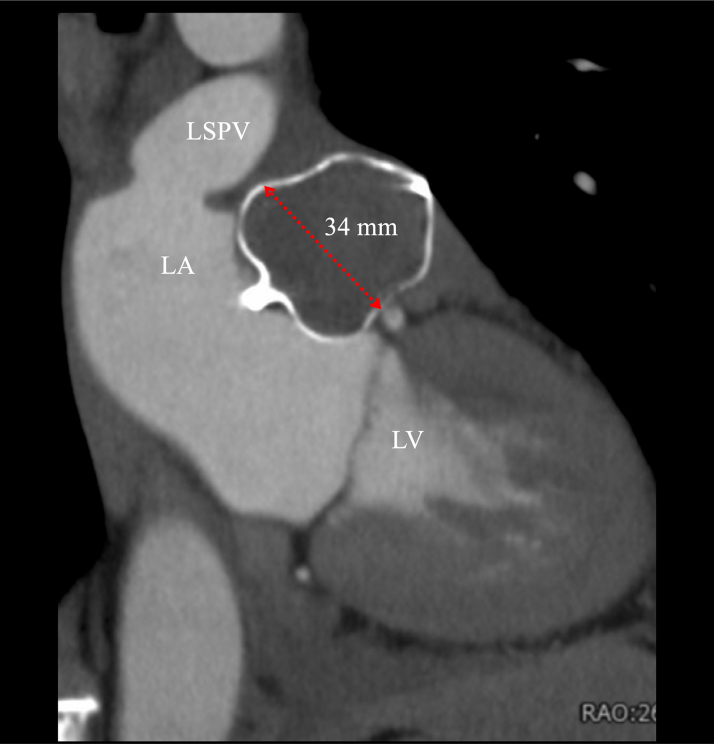
Follow-Up CT Findings Cardiac CT at 1-month follow-up showing stable device position, appropriate compression (15%), and no device-related thrombus. CT = computed tomography; LA = left atrium; LSPV = left superior pulmonary vein; LV = left ventricle.

The patient's recovery was uneventful. Cardiac CT at the 1-month follow-up confirmed a complete LAA seal without leak or device-related thrombus (Figure 7, Video 6). The maximum device size on cardiac CT was 34 mm, corresponding to an appropriate compression rate of 15%. At the 6-month follow-up, the patient remained asymptomatic with no recurrence of palpitation.

## Discussion

This case highlights 3 important findings: 1) Concomitant PFA and LAAO is feasible in patients with LAA-origin AT; 2) PFA within the LAA may cause significant edema and narrowing of the LAA orifice, complicating device sizing; and 3) prudent device selection guided by pre-PFA imaging is crucial to avoid late peridevice leak.

LAA-origin AT is rare, representing a small subset of focal ATs.^1^ Outcomes with RFCA are variable, and distal LAA foci are often resistant to RFCA.^2^^,^^3^ PFA has emerged as an attractive alternative therapy because of its myocardial selectivity, reduced risk of collateral injury, and favorable safety profile. Recent studies suggest its efficacy in targeting LAA-origin AT.^4^ On the other hand, electrical LAA isolation raises concerns about postprocedural thrombus formation and thromboembolic events. Observational studies have documented increased stroke risk after electrical LAA isolation.^5^, ^6^, ^7^ LAAO represents a rational adjunct to mitigate this risk.

Previous reports have indicated that electroporation can create acute edema at the ridge during PFA procedures for AF with LAAO.^8^^,^^9^ In the present case, PFA within the LAA induced significant tissue edema, reducing the maximal orifice diameter by 5 mm and causing swelling of the pectinate muscle. These changes complicated LAAO device sizing. One possible strategy is to delay device implantation until edema resolves, allowing accurate sizing. However, this waiting approach exposes the patient to thromboembolic risk. Alternatively, proceeding with concomitant LAAO requires careful consideration. Downsizing the device based on post-PFA imaging risks late peridevice leak once edema resolves. Rather, maintaining device size based on pre-PFA imaging, with intentional overcompression, provides a more secure LAA sealing.

Follow-up cardiac CT imaging in this case confirmed the effectiveness of the latter strategy, with no leak and appropriate device compression rate. These findings demonstrate that device sizing should rely on pre-PFA imaging rather than immediate post-PFA imaging, which may underestimate the true orifice size.

Of note, in our case, swelling of the pectinate muscle made deployment of a 40-mm device technically challenging. Thus, procedural success also depends on meticulous intraprocedural manipulation by experienced operators and high-quality intraprocedural imaging to guide deployment.

Although the Watchman FLX Pro was successfully used here, the feasibility of other LAAO devices in the context of PFA for LAA-origin AT remains to be investigated. More clinical experience will be necessary to establish standardized strategies for concomitant therapy. Recently, the OPTION trial evaluated adjunctive LAAO performed at the time of catheter ablation for atrial fibrillation and demonstrated noninferior outcomes for safety and adverse events compared with oral anticoagulation.^10^ Our experience of concomitant PFA and LAAO for LAA-origin AT aligns with these findings, supporting the feasibility and potential safety advantage of this combined approach.

## Conclusions

Concomitant PFA and LAAO for LAA-origin AT is feasible. However, careful device sizing based on pre-PFA imaging is essential to minimize the risk of late peridevice leak.

## Funding Support and Author Disclosures

The authors have reported that they have no relationships relevant to the contents of this paper to disclose.

**Take-Home Messages**

• Pulsed-field ablation for LAA–origin atrial tachycardia can cause significant tissue edema and narrowing of the orifice.
• Prudent selection of transcatheter left atrial appendage occlusion device and intentional overcompression, guided by preablation imaging findings, are crucial.

## Appendix

Video 1 TEE Before PFA TEE before PFA demonstrating an LAA maximal orifice diameter of 32 mm without thrombus.

Video 2 LAA Angiography The LAA angiography showed a large cactus-shaped LAA with a maximum orifice diameter of 32 mm.

Video 3 TEE After PFA Intraprocedural TEE showed acute edema of the pectinate muscle with ostial narrowing and contour change, reducing the maximal diameter to 27 mm.

Video 4 Intraprocedural TEE During LAAOA 40-mm device was deployed according to the preablation plan with intentional overcompression (29%), yielding stable seating.

Video 5 Intraprocedural TEE During LAAOA 40-mm device was deployed, with no peridevice leak on color Doppler.

Video 6 Follow-Up Cardiac CTCardiac CT confirmed a complete LAA seal without leak or device-related thrombus.
